# Association of Haptoglobin Phenotypes with Outcomes in Patients with Spontaneous Intracerebral Hemorrhage

**DOI:** 10.3390/life12071001

**Published:** 2022-07-06

**Authors:** Jin Pyeong Jeon, Sung Woo Han, Tae Yeon Kim, Seung Hyuk Lim, Dong Hyuk Youn, Jong Kook Rhim, Jeong Jin Park, Jun Hyong Ahn, Heung Cheol Kim, Jinseo Yang

**Affiliations:** 1 Department of Neurosurgery, Hallym University College of Medicine, Chuncheon 24253, Korea; jjp6553@hallym.or.kr; 2 Institute of New Frontier Research, Hallym University College of Medicine, Chuncheon 24253, Korea; m21082@hallym.ac.kr (S.W.H.); m22084@hallym.ac.kr (T.Y.K.); m21074@hallym.ac.kr (S.H.L.); dhyoun@hallym.ac.kr (D.H.Y.); 3 Department of Neurosurgery, Jeju National University College of Medicine, Jeju 63243, Korea; pedineur@daum.net; 4 Department of Neurology, Konkuk University Medical Center, Seoul 05030, Korea; parkjj@kuh.ac.kr; 5 Department of Neurosurgery, Kangwon National University Hospital, Chuncheon 24289, Korea; sparkahn@knuh.or.kr; 6 Department of Radiology, Hallym University College of Medicine, Chuncheon 24253, Korea; khc@hallym.or.kr

**Keywords:** intracerebral hemorrhage, haptoglobin, perihematomal edema

## Abstract

Object. We aimed to investigate the association of Haptoglobin (Hp) phenotypes with perihematomal edema (PHE) and neurological outcomes after intracerebral hemorrhage (ICH). Methods. This prospective multicenter study enrolled patients that suffered ICH from March 2017 to February 2020. Hp phenotypes were determined using Western blotting; relative α1 intensity was calculated in patients with Hp2-1. A multivariable logistic regression analysis was then conducted to identify risk factors for increased relative PHE at 96 h and 3-month poor outcomes. Results. In total, 120 patients were ultimately enrolled: Hp1-1 (n = 15, 12.5%); Hp2-1 (n = 51, 42.5%); and Hp2-2 (n = 54, 45.0%). Hp phenotype was significantly associated with PHE (*p* = 0.028). With Hp1-1 as a reference value, Hp2-2 significantly increased the likelihood of increased rPHE (OR = 6.294, 95% CI: 1.283–30.881), while Hp2-1 did not (OR = 2.843, 95% CI: 0.566–14.284). Poor outcomes were found to be closely associated with hematoma volume at admission (OR = 1.057, 95% CI: 1.015–1.101) and surgical treatment (OR = 5.340, 95% CI: 1.665–17.122) but not Hp phenotypes (*p* = 0.190). Further, a high level of relative α1 intensity was identified to be significantly associated with decreased rPHE (OR = 0.020, 95% CI: 0.001–0.358). However, the relative α1 intensity was not associated with poor outcomes (OR = 0.057, 95% CI: 0.001–11.790). Conclusions: ICH patients with Hp2-2 exhibited a higher likelihood of increased rPHE than those with Hp1-1. Higher relative α1 intensities were identified to be closely associated with rPHE in patients with Hp2-1.

## 1. Introduction

Intracerebral hemorrhage (ICH) accounts for about 15–20% of all strokes in Korea. Compared to ischemic stroke, ICH occurs at a younger age and has poorer neurological outcomes [1,2]. The following radiological risk factors are associated with poor outcomes: larger hematoma volume at admission, hemorrhage growth, the presence of intraventricular hemorrhage (IVH), and perihematomal edema (PHE) that worsens over time [3]. While a series of radiological risk factors indicates the severity of primary brain injury, PHE reflects the degree of secondary brain injury after the initial bleeding [4]. Even when initial ICH is successfully treated by the appropriate medical and surgical treatments, PHE can still induce an increased intracranial pressure (IICP) and worsen the patient’s prognosis during follow-up. PHE has different mechanisms, depending on the time that has elapsed after ICH. Immediately after bleeding, the first stage of PHE manifests cytotoxic edema due to the difference in the osmotic gradient. With a worsening neuroinflammatory response and blood–brain barrier (BBB) damage, vasogenic edema becomes a main cause of the secondary stage of PHE [4]. In the third stage of PHE, toxic free hemoglobin (Hb) derived from erythrocyte lysis in the perihematomal space stimulates reactive oxygen species (ROS) production and a further inflammatory response, typically 2–3 days after ICH. It is therefore important to selectively protect against Hb toxicity that causes cytotoxic and inflammatory responses [5].

Haptoglobin (Hp) limits free Hb toxicity by making Hp-Hb complexes and achieving removal via the cell surface receptor of CD163 [6,7]. In humans, there are three types of Hp—Hp1-1, Hp2-1, and Hp2-2—depending on the combination of alpha (α) and beta (β) chains [8,9]. Hp-Hb complex clearance varies according to the particular combination of Hp alleles [10]. Compared to ICH patients with Hp2-2, those with Hp1-1 are theoretically expected to have less PHE and better neurological outcomes. Halstead et al. [11] reported that within the first 96 h after ICH Hp1-1 significantly increased the risk of PHE compared to Hp2-2 [11]. However, the Hp 2 allele has also been closely associated with poor functional outcomes in patients with ICH [12]. We believe that these conflicting results may be attributable to the fact that the previous research did not control for several variables that could influence the outcomes, including initial hematoma volume, hematoma growth, ICH location, constricted blood pressure control in the acute phase, and the time interval between symptom onset and ICH diagnosis. Thus, to more accurately assess the outcomes of ICH patients according to Hp phenotypes, it is necessary to narrow the inclusion criteria relative to those used in previous studies. Taking these facts into account, we have aimed to answer the following two issues: First, to minimize potential factors that may affect outcomes, we investigated the association between Hp phenotypes and PHE and neurological outcomes specifically in supratentorial ICH patients with hematoma volumes less than 60 mL at admission and who were actively treated with antihypertensive medication in the acute period. Second, we evaluated risk factors associated with outcomes in patients with Hp2-1. Although the ratio of Hp2-1 patients is typically observed to be 35–50%, most of the existing studies predicting the outcome after ICH have focused on the Hp1 allele over the Hp 2 allele [7,12]. Kim et al. [9] observed various α1 intensities compared to α2 intensities in patients with Hp2-1. In particular, higher α1 intensities were identified to be linked to a lower risk of delayed cerebral ischemia following subarachnoid hemorrhage. The authors assumed that a predominant effect of the Hp dimer was dependent on the relative α1 intensity. Thus, a higher α1 intensity of Hp2-1 acts like an Hp1-1 with less oxidative stress [9]. Taken together, we investigated whether the relative α1 intensity of Hp is closely associated with ICH outcomes.

## 2. Patients and Methods

### 2.1. Patient Population

**Figure 1:**
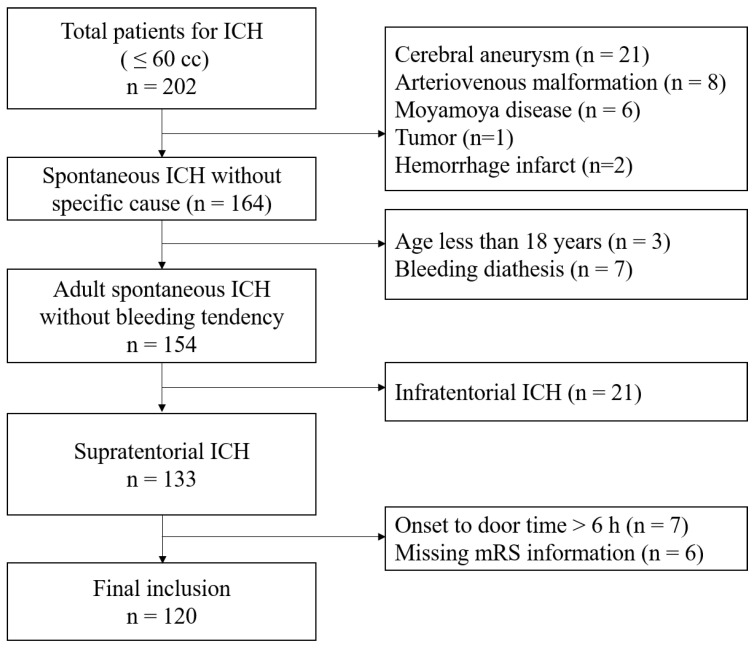
Flow chart of the study.

This study cohort was obtained from the stroke database entitled “The First Korean Stroke Genetics Association Research”; this stroke database consists of various types of prospectively collected data—including clinical, radiological, and genetic data—of patients with cerebrovascular diseases at the five university hospitals (https://1ksgh.org/, accessed on April 2022) [13,14]. From this database, we included ICH patients between March 2017 and February 2020 with the following conditions: (1) adults over 18 years of age; (2) spontaneous ICH not associated with vascular malformation; (3) ICH diagnosis within 6 h of symptom onset; (4) supratentorial ICH; (5) initial hematoma volume less than 60 mL; and (6) patients who received intensive antihypertensive treatments targeting BP < 140 mmHg in the acute period [1]. Meanwhile, we excluded patients with the following conditions: (1) traumatic ICH; (2) ICH associated with vascular malformation such as intracranial aneurysm, arteriovenous malformation, and moyamoya disease; (3) insufficient medical and radiological information; (4) previous history of ICH or cerebral infarction; and (5) patients who declined to undergo Hp phenotyping (Figure 1) [14].

### 2.2. Study Outcomes

The primary outcome in this work was to evaluate the association between Hp phenotypes and relative perihematomal edema (rPHE) at 96 h after ICH. The secondary outcome was to evaluate poor neurological outcomes at 3 months according to Hp phenotypes. We also analyzed outcomes in ICH patients with Hp2-1 based on their relative α1 intensity of Hp [7,9]. A poor neurological outcome was defined as a score of 3–6 on the modified Rankin Scale (mRS). Medical records (e.g., gender, age, hypertension, diabetes mellitus, coronary heart disease, chronic kidney disease, hyperlipidemia, history of antiplatelet and anticoagulation use, and smoking) were reviewed. Radiological records regarding hematoma location, type (lobar and deep ICH), hematoma volume, and rPHE occurring within the first 96 h after diagnosis were also reviewed [11,15,16]. The imaging protocol for hospitalized patients, especially spontaneous ICH patients with less than 60 cc, is mentioned below. First, an MRI was electively performed 1–2 days after admission to identify the cause associated with ICH. Second, a follow-up CT was checked once more within 3–4 days after ICH occurrence, considering the time of the MRI. Hematoma volume and rPHE were measured using an open-source 3D-slice software to which the original DICOM format files produced by computed tomography (CT) (3D-Slicer, Harvard University, Cambridge, MA, USA) were uploaded and examined by two neurointerventionists who each had more than 10 years of experience [17]. rPHE was defined as edema volume divided by hematoma volume. In a modification to the method used in the previous reports, an rPHE more than 1.4 at 96 h was regarded as increased PHE [11,15]. This study was approved by the institutional review boards of all of the participating hospitals (IRB No: 2016-31, 2017-113, 2018-6, and 2019-06). Informed consent was obtained from the patients or their legal representatives, as appropriate.

### 2.3. Haptoglobin Phenotyping

**Figure 2:**
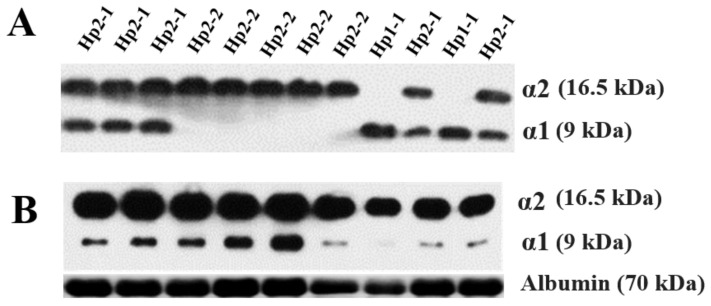
Western blotting analyses of haptoglobin (Hp) phenotype (**A**) and Hp2-1 only for assessing relative alpha 1 intensity (**B**).

Hp phenotyping was performed according to our previously described protocol [7]. First, the Hp phenotype was classified into Hp1-1, Hp2-1, and Hp2-2 using polyacrylamide gel electrophoresis followed by immunoblotting to detect α1 and α2 chains (Figure 2) [7,18]. Next, Western blotting was performed again to calculate the relative α1 intensity of Hp in patients expressing Hp2-1. A more detailed description of the method is as follows: A 1:75 dilution of serum was made by adding 1 μL of serum to 74 μL of phosphate-buffered saline. The samples were prepared by mixing a serum diluent with an equal volume of 2x-SDS sample buffer (Bio-Rad, Hercules, CA, USA) and boiled at 95 °C for 8 min. After boiling, 10 μL of each sample was loaded on 15% polyacrylamide gel and electrophoresed for 150 min at 100 V (Bio-Rad, CA, USA). After transfer, the membranes were blocked with 5% BSA in TBST (10 mM Tris-HCl pH8.0, 150 mM NaCl) including 0.01% Tween-20 for 1 h. The membranes were then incubated overnight with a polyclonal rabbit anti-human haptoglobin antibody (Dako, Glostrup, Denmark) diluted 1:10,000 in blocking buffer at 4 °C. After being washed three times with TBST, the membranes were incubated with horseradish peroxidase (HRP)-conjugated goat anti-rabbit IgG (Abcam, Cambridge, UK) at 1:10,000 for 1 h at room temperature. Following a final washing step, an HRP substrate (Thermo, Waltham, MA, USA) was added to the membrane, at which point chemiluminescence was detected using X-ray film (Kodak, Rochester, NY, USA). To determine the polymeric composition of Hp2-1 based on molecular size, additional immunoprecipitation was carried out with an anti-Hp antibody. To immunoprecipitate Hp, 0.3 mg/mL serum from patients with Hp2-1 was incubated overnight with an anti-Hp antibody at 4 °C. The immune complexes were precipitated with protein A/G Sepharose (Santa Cruz, CA, USA) and analyzed through Western blotting. An anti-albumin antibody (Abcam, Cambridge, UK) was used as a loading control. The intensities of α1 chain and albumin were measured using ImageJ software (Version 1.49v, National Institutes of Health, Bethesda, MD, USA) [7,9].

### 2.4. Statistical Analysis

Discrete and continuous data are, respectively, described as means with proportions and medians (25–75 percentile). A univariate analysis was conducted to find the relevant factors associated with certain outcomes. A multivariate analysis was conducted to identify the risk factors for certain outcomes, including variables with *p*-values less than 0.20. A relative α1 intensity correlation with outcomes was further analyzed for Hp2-1 patients only. The relative α1 intensity was calculated as the α1 intensity divided by the albumin intensity [7,9]. *p* values < 0.05 were regarded as being statistically significant. Statistical analyses were conducted using SPSS V.19 (SPSS, Chicago, IL, USA).

## 3. Results

### 3.1. Primary Outcomes

In total, 120 patients were ultimately included in the analysis after exclusion (Figure 1). Hp1-1 was noted in 15 patients (12.5%), Hp2-1 was noted in 51 patients (42.5%), and Hp2-2 was noted in 54 patients (45.0%). Increased PHE—defined as rPHE ≥ 1.4 at 96 h—was observed significantly more often in patients with Hp2-2 (n = 26, 48.1%) than those with Hp2-1 (n = 15, 29.4%) or Hp1-1 (n = 2, 13.3%) (*p* = 0.020) (Table 1 and Table 2). The multivariate analysis revealed that Hp2-2 significantly increased the likelihood of increased rPHE (OR = 6.294, 95% CI: 1.283–30.881) when Hp1-1 was used as a reference value. Other variables such as hypertension, coronary artery disease, drug medication with antiplatelet or anticoagulating agents, and hematoma volume at admission led to no significant increase in rPHE (Table 3). The Nagelkerke R-Square value was 0.131.

Poor outcomes were observed in 50 patients (41.7%) with ICH: Hp1-1, n = 2 (13.3%); Hp2-1, n = 22 (43.1%); and Hp2-2, n = 26 (48.1%) (Table 4). Nine patients (7.5%) died during hospitalization. Three patients died due to neurological damage, and six patients died due to pneumonia and thromboembolic complications. The multivariate analysis showed that higher Hb levels (OR = 1.447, 95% CI: 1.115–1.877) and hematoma volumes at admission (OR = 1.057, 95% CI: 1.015–1.101) and having received surgical treatments consisting of either burr-hole trephination or craniotomy (OR = 5.340, 95% CI: 1.665–17.122) increased the risk of poor outcomes after ICH. When Hp1-1 was used as a reference value, Hp2-2 (OR = 4.286, 95% CI: 0.733–25.048) and Hp2-1 (OR = 5.265, 95% CI: 0.881–31.457) did not significantly increase the likelihood of poor outcomes (Table 5). The Nagelkerke R-Square value was 0.338. 

### 3.2. Outcomes in Haptoglobin 2-1

We analyzed the outcomes of ICH patients with Hp2-1. Increased rPHE was observed in 15 patients (29.4%) among those with Hp2-1 (Supplemental Table S1). The multivariate analysis revealed that a higher relative α1/albumin intensity significantly decreased the risk of increased rPHE (OR = 0.020, 95% CI: 0.001–0.358) (Supplemental Table S2). The Nagelkerke R-Square value was 0.209. Poor outcomes were observed in 22 patients (43.1%) among ICH patients with Hp2-1. Relative α1/albumin intensity was not related to outcomes in ICH patients with Hp2-1 (*p* = 0.856) (Supplemental Table S3). Among the various possible risk factors, only the presence of surgical treatment significantly increased the risk of poor neurological outcomes at 3 months (OR = 6.239, 95% CI: 1.054–36.928) (Supplemental Table S4). The Nagelkerke R-Square value was 0.179.

## 4. Discussion

**Figure 3:**
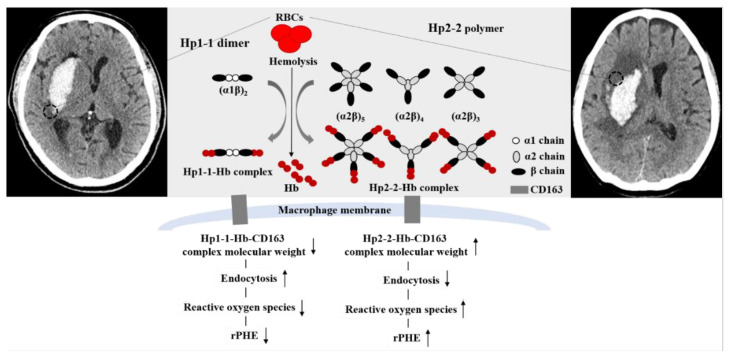
Proposed mechanism of increased relative perihematomal edema (rPHE) after primary intracerebral hemorrhage (ICH) according to haptoglobin (Hp) phenotypes (Hp1-1 vs. Hp2-2). Black dotted circles indicate PHE.

The influence of Hp phenotypes on outcomes in ICH patients has not been well-investigated compared to the same influence in patients with subarachnoid hemorrhage (SAH). Gaastra et al. [8] reported that the Hp1 allele showed a stronger protective effect against cerebral vasospasm following SAH than the Hp2 allele. On the other hand, the Hp2 allele has been reported to be associated with favorable long-term outcomes in patients with high-volume SAH [19]. ICH refers to the bleeding that occurs within the brain parenchyma; it is therefore more important to eliminate free Hb toxicity to adjacent brain cells around an ICH. In terms of protein and lipid oxidation, Hp1-1 exhibited better anti-oxidant activity than Hp2-2 [8,20]. An Hp1-1-Hb complex binding to CD163 also exhibited a better anti-inflammatory response than the corresponding Hp2-2-Hb complex [21]. Accordingly, we hypothesized that Hp1-1 may be associated with lower PHE and better outcomes than Hp2-2. However, previous studies have shown conflicting results [11,12]. Halsted et al. [11] reported that Hp1-1 was associated with a higher likelihood of PHE than Hp2-2, as measured within 96 h after onset, while Murthy et al. [12] showed that ICH patients with Hp2-2 and Hp2-1 experienced poor outcomes more frequently than those with Hp1-1. In actual clinical practice, a patient’s prognosis is largely dependent on the initial hematoma volume, hematoma growth, and location. An ICH volume exceeding 60 mL exhibited a higher mortality of approximately 90% in deep-located ICH and 70% in lobar ICH [22]. A large hematoma volume is itself closely related to increased PHE. There is also a tendency toward an increased ICH amount within the first 3 h in up to two thirds of ICH patients [23]. Regarding ICH location, patients with brainstem ICH and cerebellar ICH showed different prognoses than those with supratentorial ICH [24]. Therefore, the clinical significance of Hp phenotypes on ICH outcomes should be evaluated while controlling such factors that can affect prognosis. In this study, we have only enrolled supratentorial ICHs under 60 mL and evaluated the association of Hp phenotypes with outcomes based on relative rPHE values. Our study revealed that Hp2-2 significantly increased PHE more than Hp1-1, but Hp phenotypes were not associated with the patient’s outcome. For the first time, we identified risk factors for PHE and outcomes in ICH patients with Hp2-1. In simple terms, Hp2-1 consists of one Hp1 allele and one Hp2 allele. Thus, in the case of Hp2-1, it was mainly used for the comparative analysis of Hp1-1 vs. Hp2-1 and Hp2-2 or of Hp1-1 and Hp2-1 vs. Hp2-2. The results of a meta-analysis revealed that Hp2-2 patients had a significantly higher likelihood of poor outcomes than Hp1-1 patients following SAH (OR = 2.37, 95% CI: 1.12–5.04) [8]. However, this association was not clear when analyzing between Hp2-1 with Hp2-2 and Hp1-1 (OR = 1.50, 95% CI: 0.80–2.82) [8]. Hp2-1 structures can vary from similarity to dimer to polymer based on the α and β chain combinations. [7,9]. Han et al. [7] proposed that a relatively high α1 intensity acts in a similar manner to an Hp1-1 dimer and that it was associated with 6-month outcomes after SAH. Based on this hypothesis, we have further analyzed the outcomes in the 51 ICH patients expressing Hp2-1. Our results revealed that a higher relative α1/albumin intensity significantly decreased the likelihood of rPHE in patients with Hp2-1. We presumed that the lower molecular weight of the Hp2-1-Hb complex, which appears in the form of the high relative α1 intensity, may be associated with better clearance and reduced ROS production than the Hp2-1-Hb complex with a lower relative α1 intensity in the perihematomal lesions (Figure 3) [7,9].

In addition to the Hp studies conducted so far, future studies on the role of Hp in stroke occurrence and treatment are required in patients with hematological disease. The frequency of hematological disease as a cause of stroke is approximately 1.3%. Arboix et al. [25] reported that patients who were young and had a prior history of venous thrombosis and recurrent stroke of undetermined cause were suspicious of hematological disease for stroke. Moreover, Hp 2 alleles are more disadvantageous than Hp1 alleles in sickle cell disease [26]. When considering that Hp is essential for reducing blood toxicity, oxidative stress, and inflammation in sickle cell disease, it is hypothesized that research on Hp is necessitated to find the clue and treatment for stroke, including ICH, in various hematologic diseases in the future.

This study has some limitations. First, we measured PHE at 96 h after ICH diagnosis while following a previous study [11]. PHE develops within 24 h and may persist for several weeks [3,27]. Thus, our results are limited in their ability to reflect the association between Hp phenotypes and PHE in the acute period. Second, we did not analyze Hp-Hb binding capacity or affinity to CD163 of the macrophage. Although Hp1-1 showed a better anti-oxidant effect than Hp2-2, the interaction with CD163 was higher in Hp2-2 than Hp1-1 in an in vitro study [8,28]. Therefore, it is necessary to comprehensively analyze the differences in Hp-Hb complex clearance and ROS production throughout the entire process. Third, we enrolled patients who visited the hospital within 6 h of symptom onset, were diagnosed with supratentorial ICH, and who received active anti-hypertensive treatment at the same time. Accordingly, our results may differ from those considering patients with large hematomas, infratentorial ICH, delayed visits to the emergency room, or those without active antihypertensive treatment. Moreover, it seems that the patient’s in-hospital mortality rate was relatively low due to these specific inclusion criteria. Nevertheless, it should be noted that the rate of hemorrhagic lacunar stroke was relatively high in our study. Hemorrhagic lacunar stroke accounted for 3.8% of all lacunar syndrome and 7.4% of ICH [29]. In the present study, we did not consider hemorrhagic lacunar stroke in the analysis. Moreover, the inclusion criteria were specified only for ICH amount less than 60 cc, and the minimum amount of hemorrhage was not considered. Arboix et al. [29] reported that patients with hemorrhagic lacunar stroke were more likely to have a gradual onset of symptoms and involvement of the internal capsule compared with those with non-lacunar ICH. Thus, there is a possibility that deep-located ICH did not significantly increase the risk of poor outcomes compared to lobar ICH in our study. Lastly, our results may be underestimated due to the small number of patients. Unfortunately, we did not calculate the sample size at the initiation of the study. A power analysis for the sample size suggested that at least 158 participants are required to confirm our hypothesis. Therefore, caution is taken with these limitations when interpreting the results of our study.

## 5. Conclusions

ICH patients with Hp2-2 showed a higher likelihood of increased rPHE than those with Hp1-1. In addition, a higher relative α1 intensity was identified to be closely associated with a lower risk of rPHE ≥ 1.4 in patients with Hp2-1.

## Figures and Tables

**Table 1:** Baseline characteristics of enrolled patients with intracerebral hemorrhage according to haptoglobin (Hp) phenotype.

Variables	Hp1-1 (n = 15)	Hp2-1 (n = 51)	Hp2-2 (n = 54)	*p*-Value
Clinical variables				
Male	6 (40.0%)	29 (56.9%)	30 (55.6%)	0.496
Age (years)	64.3 ± 10.7	62.9 ± 16.1	65.6 ± 13.8	0.674
Hypertension	11 (73.3%)	34 (66.7%)	27 (50.0%)	0.116
Diabetes mellitus	3 (20.0%)	10 (19.6%)	8 (14.8%)	0.782
Coronary artery disease	1 (6.7%)	5 (9.8%)	4 (7.4%)	0.878
Hyperlipidemia	2 (13.3%)	7 (13.7%)	8 (14.8%)	0.982
Chronic kidney disease	2 (13.3%)	3 (5.9%)	2 (3.7%)	0.371
Smoking	2 (13.3%)	10 (19.6%)	10 (18.5%)	0.858
Antiplatelet or anticoagulation	4 (26.7%)	11 (21.6%)	10 (18.5%)	0.778
Laboratory variables				
Albumin (g/L)	4.2 ± 0.4	4.3 ± 0.5	4.1 ± 0.4	0.159
Hemoglobin (g/dL)	12.9 ± 1.9	13.6 ± 2.2	13.9 ± 1.6	0.247
Platelet (×10^9^/L)	249.7 ± 89.3	235.3 ± 67.8	236.8 ± 82.2	0.949
Radiologic variables				
Deep ICH	12 (80.0%)	47 (92.1%)	44 (81.5%)	0.230
Hematoma volume at admission (cc)	14.2 ± 9.7	17.7 ± 11.9	20.3 ± 13.5	0.135
rPHE ≥ 1.4 at 96 h	2 (13.3%)	15 (29.4%)	26 (48.1%)	0.020
Treatment				
Burr-hole trephination or craniotomy	3 (20.0%)	8 (15.7%)	13 (24.1%)	0.562
Outcome				
Poor neurological outcomes at 3 months	2 (13.3%)	22 (43.1%)	26 (48.1%)	0.051

rPHE indicates relative perihematomal edema.

**Table 2:** Univariate analysis of relevant factors associated with increased relative perihematomal edema (rPHE), defined as rPHE ≥ 1.4 at 96 h, after intracerebral hemorrhage.

Variables	rPHE < 1.4 (n = 77)	rPHE ≥ 1.4 (n = 43)	*p*-Value
Clinical variables			
Male	34 (44.2%)	21 (48.8%)	0.622
Age, years	67.0 (50.0–74.3)	68.0 (53.3–75.8)	0.760
Hypertension	50 (64.9%)	22 (51.2%)	0.140
Diabetes mellitus	14 (18.2%)	7 (16.3%)	0.793
Coronary artery disease	9 (11.7%)	1 (2.3%)	0.075
Hyperlipidemia	12 (15.6%)	5 (11.6%)	0.551
Chronic kidney disease	4 (5.2%)	3 (7.0%)	0.690
Smoking	15 (19.5%)	7 (16.3%)	0.664
Antiplatelet or anticoagulation	19 (24.7%)	6 (14.0%)	0.166
Laboratory variables			
Albumin (g/L)	4.3 (3.9–4.5)	4.3 (4.0–4.5)	0.462
Hemoglobin (g/dL)	13.6 (12.7–14.6)	13.6 (12.6–15.1)	0.763
Platelet (×10^9^/L)	239 (191–287)	234 (186–305)	0.852
Radiologic variables			
Deep ICH	65 (84.4%)	38 (88.4%)	0.551
Hematoma volume at admission (cc)	13.6 (8.8–22.7)	15.0 (11.3–26.0)	0.109
Burr-hole trephination or craniotomy	15 (19.5%)	9 (20.9%)	0.849
Haptoglobin phenotypes			0.020
Hp1-1	13 (16.9%)	2 (4.6%)	
Hp2-1	36 (46.8%)	15 (34.9%)	
Hp2-2	28 (36.3%)	26 (60.5%)	

**Table 3:** Multivariate logistic regression analysis to identify the risk factors of increased relative perihematomal edema (rPHE), defined as rPHE ≥ 1.4 at 96 h, after intracerebral hemorrhage.

	Odds Ratio	95% Confidence Interval	*p*-Value
Hypertension	0.866	0.368–2.036	0.742
Coronary artery disease	0.172	0.020–1.440	0.104
Antiplatelet or anticoagulation	0.592	0.192–1.825	0.362
Hematoma volume at admission (cc)	1.013	0.982–1.045	0.417
Haptoglobin phenotypes			0.028
Hp1-1		1	
Hp2-1	2.843	0.566–14.284	0.204
Hp2-2	6.294	1.283–30.881	0.023

**Table 4:** Univariate analysis of relevant factors associated with poor outcomes after intracerebral hemorrhage.

Variables	Good Outcomes (n = 70)	Poor Outcomes (n = 50)	*p*-Value
Clinical variables			
Male	35 (50%)	30 (60.0%)	0.278
Age, years	69.5 (53.0–76.0)	63.0 (50.0–74.0)	0.138
Hypertension	47 (67.1%)	25 (50.0%)	0.059
Diabetes mellitus	14 (20.0%)	7 (14.0%)	0.394
Coronary artery disease	7 (10.0%)	3 (6.0%)	0.434
Hyperlipidemia	10 (14.3%)	7 (14.0%)	0.965
Chronic kidney disease	5 (7.1%)	2 (4.0%)	0.469
Smoking	11 (15.7%)	11 (22.0%)	0.380
Antiplatelet or anticoagulation	17 (24.3%)	8 (16.0%)	0.271
Laboratory variables			
Albumin (g/L)	4.3 (4.0–4.5)	4.3 (4.0–4.6)	0.659
Hemoglobin (g/dL)	13.3 (12.3–14.6)	14.1 (13.3–15.4)	0.005
Platelet (×10^9^/L)	245 (196–297)	229 (189–300)	0.497
Radiologic variables			
Deep ICH	59 (84.3%)	44 (88.0%)	0.565
Hematoma volume at admission (cc)	12.0 (8.8–18.0)	19.0 (12.0–30.0)	0.001
rPHE ≥ 1.4 at 96 h	22 (31.4%)	21 (42.0%)	0.234
Burr-hole trephination or craniotomy	6 (8.6%)	18 (36.0%)	<0.001
Haptoglobin phenotypes			0.051
Hp1-1	13 (18.6%)	2 (4.0%)	
Hp2-1	29 (41.4%)	22 (44.0%)	
Hp2-2	28 (40.0%)	26 (52.0%)	

rPHE indicates relative perihematomal edema.

**Table 5:** Multivariate logistic regression analysis of risk factors associated with poor outcomes after intracerebral hemorrhage.

	Odds Ratio	95% Confidence Interval	*p*-Value
Hypertension	0.433	0.181–1.309	0.061
Age, years	0.994	0.962–1.027	0.717
Hemoglobin	1.447	1.115–1.877	0.005
Hematoma volume at admission (cc)	1.057	1.015–1.101	0.007
Burr-hole trephination or craniotomy	5.340	1.665–17.122	0.005
Haptoglobin phenotypes			0.190
Hp1-1		1	
Hp2-1	5.265	0.881–31.457	0.069
Hp2-2	6.294	1.283–30.881	0.106

## Data Availability

The data that support the findings of this study were submitted as online supplemental material, and further detailed information is available upon request to the corresponding author. All genotype and phenotype resources are managed by “The First Korean Stroke Genetics Association Research” study constructed from the Sacred Heart Hospital Stroke Database (https://1ksgh.org/, accessed on April 2022).

## Supplementary Materials

The following supporting information can be downloaded at: https://www.mdpi.com/article/10.3390/life12071001/s1, Table S1. Univariate analysis of relevant factors associated with increased relative perihematomal edema (rPHE), defined as rPHE ≥ 1.4 at 96 h, after intracerebral hemorrhage in patients with haptoglobin 2-1. Table S2. Multivariate logistic regression analysis of increased relative perihematomal edema (rPHE), defined as rPHE ≥ 1.4 at 96 h, after intracerebral hemorrhage in patients with haptoglobin 2-1. Table S3. Univariate analysis of relevant factors associated with poor outcomes after intracerebral hemorrhage in patients with haptoglobin 2-1. Table S4. Multivariate logistic regression analysis of poor outcomes after intracerebral hemorrhage in patients with haptoglobin 2-1.
