# Supplementary material for: Association of Haptoglobin Phenotypes with Outcomes in Patients with Spontaneous Intracerebral Hemorrhage
Source: Life (Basel). 2022 Jul 6;12(7):1001. doi: 10.3390/life12071001 (PMC9318044; doi:10.3390/life12071001)
Supplement: Supplementary file 1 [file life-12-01001-s001.zip › life-1732306-supplementary.pdf]

**SUPPLEMENTAL DATA**

**Supplemental Table S1.** Univariate analysis of relevant factors associated with increased relative perihematomal edema (rPHE), defined as rPHE ≥ 1.4 at 96 hours, after intracerebral hemorrhage in patients with haptoglobin 2-1.

| Variables | rPHE < 1.4 (n = 36) | rPHE ≥ 1.4 (n = 15) | p-value |
|---|---|---|---|
| Clinical variables | | | |
| Male | 22 (61.1%) | 7 (46.7%) | 0.343 |
| Age, years | 67.0 (49.0-75.5) | 62.0 (50.5-74.5) | 0.804 |
| Hypertension | 25 (69.4%) | 9 (16.0%) | 0.514 |
| Diabetes mellitus | 5 (13.9%) | 5 (33.3%) | 0.111 |
| Coronary artery disease | 3 (8.3%) | 2 (13.3%) | 0.584 |
| Hyperlipidemia | 2 (5.6%) | 2 (13.3%) | 0.958 |
| Chronic kidney disease | 2 (5.6%) | 2 (13.3%) | 0.347 |
| Smoking | 7 (19.4%) | 5 (33.3%) | 0.964 |
| Antiplatelet or anticoagulation | 6 (16.7%) | 5 (33.3%) | 0.776 |
| Laboratory variables | | | |
| Albumin (g/L) | 4.3 (4.1-4.7) | 4.3 (4.2-4.6) | 0.640 |
| Hemoglobin (g/dl) | 13.9 (12.5-14.9) | 12.6 (10.6-14.1) | 0.061 |
| Platelet (×$10^9$/L) | 242 (198-267) | 234 (200-296) | 0.649 |
| Radiologic variables | | | |
| Deep ICH | 33 (91.7%) | 14 (93.3%) | 0.840 |
| Hematoma volume at admission (cc) | 13.0 (8.4-22.2) | 20.0 (11.3-25.5) | 0.085 |
| Burr-hole trephination or craniotomy | 5 (13.9%) | 5 (33.3%) | 0.585 |
| Haptoglobin | | | 0.025 |
| Relative $\alpha1$ /albumin intensity | 0.325 (0.225-0.435) | 0.210 (0.130-0.255) | |

**Supplemental Table S2. Multivariate logistic regression analysis of increased relative perihematomal edema (rPHE), defined as rPHE ≥ 1.4 at 96 hours, after intracerebral hemorrhage in patients with haptoglobin 2-1.**

| | Odds ratio | 95% Confidence interval | p-value |
|---|---|---|---|
| Diabetes mellitus | 3.136 | 0.589-16.705 | 0.181 |
| Hemoglobin | 0.768 | 0.544-1.085 | 0.134 |
| Hematoma volume at admission (cc) | 1.050 | 0.993-1.110 | 0.088 |
| Relative $\alpha$1 /albumin intensity | 0.002 | 0.001-0.358 | 0.020 |

**Supplemental Table S3.** Univariate analysis of relevant factors associated with poor outcomes after intracerebral hemorrhage in patients with haptoglobin 2-1.

| Variables | Good outcome (n = 29) | Poor outcome (n = 22) | p-value |
|---|---|---|---|
| Clinical variables | | | |
| Male | 15 (51.7%) | 14 (63.6%) | 0.395 |
| Age, years | 71.0 (51.3-76.7) | 54.0 (49.0-70.0) | 0.142 |
| Hypertension | 22 (75.9%) | 12 (54.5%) | 0.110 |
| Diabetes mellitus | 8 (27.6%) | 2 (9.1%) | 0.099 |
| Coronary artery disease | 4 (13.8%) | 1 (4.5%) | 0.271 |
| Hyperlipidemia | 5 (17.2%) | 2 (9.1%) | 0.402 |
| Chronic kidney disease | 2 (6.9%) | 2 (9.1%) | 0.773 |
| Smoking | 6 (20.7%) | 4 (18.2%) | 0.823 |
| Antiplatelet or anticoagulation | 5 (17.2%) | 4 (18.2%) | 0.930 |
| Laboratory variables | | | |
| Albumin (g/L) | 4.3 (4.1-4.6) | 4.3 (4.2-4.6) | 0.468 |
| Hemoglobin (g/dl) | 13.0 (12.1-14.9) | 13.9 (12.9-14.6) | 0.499 |
| Platelet ($\times 10^9$/L) | 239 (196-274) | 252 (200-300) | 0.549 |
| Radiologic variables | | | |
| Deep ICH | 26 (89.7%) | 21 (95.4%) | 0.445 |
| Hematoma volume at admission (cc) | 13.0 (10.0-21.0) | 16.0 (10.0-24.0) | 0.401 |
| rPHE ≥ 1.4 at 96 hours | 8 (27.6%) | 7 (31.8%) | 0.743 |
| Burr-hole trephination or craniotomy | 2 (6.9%) | 6 (27.3%) | 0.048 |
| Haptoglobin | | | 0.856 |
| Relative $\alpha$1 /albumin intensity | 0.310 (0.193-0.403) | 0.285 (0.210-0.440) | |

rPHE indicates relative perihematomal edema

**Supplemental Table S4.** Multivariate logistic regression analysis of poor outcomes after intracerebral hemorrhage in patients with haptoglobin 2-1.

| | Odds ratio | 95% Confidence interval | p-value |
|---|---|---|---|
| Hypertension | 0.316 | 0.089-1.115 | 0.073 |
| Age, years | 0.983 | 0.944-1.023 | 0.397 |
| Diabetes mellitus | 0.394 | 0.063-2.454 | 0.318 |
| Burr-hole trephination or craniotomy | 6.239 | 1.054-36.928 | 0.044 |
